# Analysis of CFTR Gene Mutations in Children with Cystic Fibrosis, First Report from North-East of Iran

**Published:** 2013-08

**Authors:** Atieh Mehdizadeh Hakkak, Mohammad Keramatipour, Saeid Talebi, Azam Brook, Jalil Tavakol Afshari, Amin Raazi, Hamid Reza Kianifar

**Affiliations:** 1 Clinic of Cystic Fibrosis, Mashhad University of Medical Sciences, Mashhad, Iran; 2 Department of Medical Genetics, Tehran University of Medical Sciences, Tehran, Iran; 3 Department of Medical Genetics, Tehran University of Medical Sciences, Tehran, Iran; 4 Department of Medical Genetics, Tehran University of Medical Sciences, Tehran, Iran; 5 Bu-Ali Research Institute, Department of Immunogenetic & Tissue Cultlure, Mashhad University of Medical Sciences, Mashhad, Iran; 6 Clinic of Cystic Fibrosis, Mashhad University of Medical Sciences, Mashhad, Iran; 7 Department of Pediatrics, Mashhad University of Medical Sciences, Mashhad, Iran

**Keywords:** CFTR, Cystic Fibrosis, Mutation, Sequencing, PCR

## Abstract

***Objective(s):*** More than 1500 registered mutations in cystic fibrosis transmembrane regulator (CFTR) gene are responsible for dysfunction of an ion channel protein and a wide spectrum of clinical manifestations in patients with cystic fibrosis (CF). This study was performed to investigate the frequency of a number of well-known CFTR mutations in North Eastern Iranian CF patients.

***Material and Methods:*** A total number of 56 documented CF patients participated in this study. Peripheral blood was obtained and DNA extraction was done by the use of routin methods. Three steps were taken for determining the target mutations: ARMS-PCR was performed for common CFTR mutations based on previous reports in Iran and neighboring countries. PCR-RFLP was done for detection of R344W and R347P, and PCR-Sequencing was performed for exon 11 in patients with unidentified mutation throughout previous steps. Samples which remained still unknown for a CFTR mutation were sequenced for exon 12.

***Results: ***Among 112 alleles, 24 mutated alleles (21.42%) were detected: ΔF508 (10.71%), 1677delTA (3.57%), S466X (3.57%), N1303K (0.89%), G542X (0.89%), R344W (0.89%), L467F (0.89%). Eight out of 56 individuals analyzed, were confirmed as homozygous and eight samples showed heterozygous status. No mutations were detected in exon 12 of sequenced samples.

***Conclusion:*** Current findings suggest a selected package of CFTR mutations for prenatal, neonatal and carrier screening along with diagnosis and genetic counseling programs in CF patients of Khorasan.

## Introduction

Cystic Fibrosis (CF) is the most common autosomal recessive disorder in Caucasian populations (1), caused by mutation in cystic fibrosis transmembrane conductance regulator (CFTR) gene. (2, 3) The CFTR gene is a member of the ATP-binding cassette transporter gene super family which extends approximately 190 kb on chromosomal region 7q31.2 (4), and contains 27 exons. (5) The product of CFTR gene is a chloride channel protein of 1480 amino acids. The main function of CFTR protein is to maintain the hydration of secretions within airways and ducts through the transport of chloride and inhibition of sodium uptake. (6) CFTR is expressed largely in epithelial cells of airways, the gastrointestinal tract (including the pancreas and biliary system), the sweat glands, and the genitourinary system.

The oldest and most common mutation identified in CFTR gene is ΔF508 with a frequency of 66% worldwide. (7) More than 1500 mutations in the CFTR gene have been identified. (8) These include missense, frameshift, splice site, nonsense and deletion mutations. Although, the prevalence and types of mutations vary in different populations based on their geographic and ethnic origins (9, 10), a few mutations (p.F508del, p.G542X, p.N1303K, p.G551D, p.W1282X) have higher frequencies than others. Regarding both nature and location of CFTR gene mutations, the common consequence is disruption of CFTR protein function by means of different mechanisms which leads to various phenotypic manifestations (11, 12). Accurate identification of CF mutations results in more applicable programs for prevention, diagnosis, and treatment of CF.

Previous studies on Iranian CF patients have reported the type and frequency of some common CFTR mutations (13-16). Different Iranian populations each originating from different geography and ethnicity together with polymorphism of CFTR gene leads to variety of CFTR mutations in frequency and distribution (13). This study focuses on detecting 15 CFTR mutations in CF patients of Khorasan province, North- East of Iran in order to carry out more effective diagnostic and medical care services. Since Khorasan is a vast province (including North, Razavi and South Khorasan), several ethnic populations are found which may increase the variety of CFTR mutations. No data are available on genotypic characterization of Khorasanian CF patients. Therefore, this study conducted to recognize common CFTR gene mutations in 60 CF patients from North-East of Iran.

## Material and Method

***Ethical clearance***

This study was approved by Ethics Committee of Mashhad University of Medical Sciences. Informed written consent was obtained from parents after a session of counseling regarding genetic testing of the disease.

***Selection of patients***

A total of 56 unrelated families with at least one affected child with CF who attended the CF center of Dr Sheikh Pediatric Hospital in Mashhad were enrolled in this study. Cystic fibrosis was diagnosed based on following: elevated sweat chloride levels (>60 meq/l) on two occasions using pilocarpine iontophoresis method; clinical features of recurrent or persistent respiratory symptoms such as cough, difficult and shortened breathing or sputum production or evidences of malabsorption such as poor growth or chronic diarrhea; familial history of CF. Demographic and clinical information of each patient including age, sex, medical and familial history, age of disease presentation onset, growth indicators and the percentiles, CF complications (if existed), first symptoms and signs, fecal lipids and trypsin activity, pancreatic insufficiency, amount of sweat chloride and consanguinity of parents were recorded.

***Mutation analysis***

Peripheral blood was obtained from all affected children. Genomic DNA was extracted from leukocytes using DNP™ Kit (High yield DNA Purification Kit). CFTR amplification was carried out in a volume of 25 µl using genomic DNA (100 ng), forward and reverse primers (10 pM each), PCR Buffer (10X, Genet Bio Company), dNTP mixture (0.2 mM each, Roche Company) and Taq DNA polymerase (one unit, Roche Company). PCR was performed in a thermocycler which was arranged uniformly for all reactions as followed: initial denaturation step at 95°C for five min, denaturation step at 95°C for 30 sec, annealing step at 60°C for 30 sec, polymerization step at 72°C for one minute and final extension step at 72°C for seven min. PCR products were then revealed on Agarose gel through electrophoresis.

To detect the target mutations, three steps were taken:

The first step was performing amplification refractory mutation system assay (ARMS-PCR) detecting common CFTR mutations based on previous reports in Iran and neighboring countries (p.Phe508del, p.Gly542X, p.Asn1303Lys) in all DNA samples following description of Ferrie* et al* (17).

In the second step, restriction fragment length polymorphism technique (PCR-RFLP) was used to investigate p.Arg1303Lys and p.Arg347Pro mutations in exon eight. Restriction enzymes which were used for PCR-RFLP step was MspΙ and AviΙΙ. 

The third step was performing PCR-Sequencing for exon 11 in patients whose CFTR mutation was still unidentified. Patients, in whom no mutation could be detected in exon 11, were analyzed for exon 12 by PCR-Sequencing technique and compared with the CFTR reference genomic sequence. Primers used in this study are presented in Table 1.

## Results

***Demographic, clinical, and family history of CF patients***

A total of 56 unrelated CF patients from Khorasan province (35 males; mean age 24.91 months, between one month to 12 years) were enrolled in this study and were probed for CFTR gene mutations. Among 56 patients (112 alleles), 24 mutated alleles were detected (Table 2).

***Identification of mutations***

Performing ARMS-PCR for four common CFTR mutations resulted in identification of 14 mutated chromosomes: p.Phe508del in 12 chromosomes (five homozygote and two heterozygote patients), p.Gly542X in one chromosome and p.Asn1303Lys in one chromosome as well. Sequencing of exon 11 also confirmed p.Phe508del mutations.

**Table 1:** Primers used for PCR-RFLP and Sesquencing

Exon amplified	Primer sequence
8	Forward: AGA CCA TGC TCA GAT CTT CCA T Reverse: GCA AAG TTC ATT AGA ACT GAT C
11	Forward: GCA GAG TAC CTG AAA CAG GA Reverse: CAT TCA CAG TAG CTT ACC CA
12	Forward: CAA CTG TGG TTA AAG CAA TAG TGT Reverse: GCA CAG ATT CTG AGT AAC CAT AAT

**Table 2:** Demographic, clinical, and family characterizations of patients with specific CFTR mutation

No of patients	Sex	Sweat chloride (meq/lit)	Pancreatic insufficiency	Age of clinical presentation onset (month)	First clinical symptom/sign	Consanguinity of parents	Mutation status
1	M	110	+	6	Steatorrhea/Hepatomegaly	First cousin	ΔF508/ ΔF508
2	M	115	+	5	Steatorrhea/Cough/ Hepatomegaly	First cousin	ΔF508/ ΔF508
3	F	130	+	2	Steatorrhea/Cough Wheezing/Skin rash	First cousin	ΔF508/ ΔF508
4	F	180	+	1	Steatorrhea/Cough/Vomiting/Edema/Hepatomegaly	First cousin	ΔF508/ ΔF508
5	M	93	+	3.5	FTT/Steatorrhea	First cousin once removed	ΔF508/ ΔF508
6	M	100	+	At birth	Wheezing/Meconium ileus	-	ΔF508/U^*^
7	M	115	+	2	Steatorrhea/Cough/Fever	First cousin once removed	ΔF508/U
8	M	90	+	6	Cough/Wheezing	-	N1303K/U
9	F	70	+	At birth	Meconium ileus/Crackle	First cousin	G542X/U
10	F	80	-	5	Cough/Wheezing/Fever	-	R334W/U
11	M	109	+	1	Fever/Wheezing/Cough	Second cousin	S466X/ S466X
12	M	120	+	10	Cough/Wheezing/Steatorrhea	-	S466X/U
13	M	100	+	At birth	Wheezing/Meconium ileus	First cousin	S466X/U
14	M	100	+	5.5	Rectal prolapse/Cough/ Wheezing/Steatorrhea	First cousin	1677delTA/ 1677delTA
15	M	85	+	3	FTT/Sreatorrhea/Wheezing/ Cough	First cousin	1677delTA/ 1677delTA
16	F	93	+	4	Steatorrhea	-	1531C/T (L467F)/U

^* ^Unknown mutation

PCR-RFLP was operated for identification of p.Arg334Trp and p.Arg347Pro mutations and revealed only one heterozygote status for p.Arg334Trp mutation.

For detection of other mutations, sequencing was done for those patients whose one or both CFTR alleles remained incompletely identified. Sequencing data indicated nine mutated chromosomes. p.Tyr515X was detected in four chromosomes (two homozygotes), p.Ser466X also in four chromosomes (one homozygote and two heterozygotes) and p.Leu467Phe in one chromosome (Table 3).

Totally, eight out of 56 individuals analyzed were, confirmed as homozygous and eight samples showed heterozygous status. No mutations were detected in exon 12 of sequenced samples. 

## Discussion

In current study, 24 mutated alleles were detected in 16 patients out of 56 unrelated CF patients from Khorasan province in North-East of Iran. We initially detected three of common CFTR mutations among Iranian CF patients which were identified in previous studies (13) (p.Phe508del, p.Asn1303Lys, and p.Gly542X) using ARMS-PCR. Exon eight was probed for p.Arg334Trp and p.Arg347Pro mutations by PCR-RFLP which revealed only one p.Arg334Trp mutation. At final step we focused on PCR-Sequencing for exon 11 in patients who remained incompletely identified for CFTR mutation in any of CFTR alleles, resulting in identification of nine other mutated chromosomes (p.Ser466X, p.Tyr515X, and p.Leu467Phe) in addition to p.Phe508del mutation. For still unidentified samples, sequencing was done for exon 12.

**Table 3:** Mutations identified in CF children of North-East of Iran. Total chromosomes: 100%, known mutations: 21.42%, unknown mutations: 78.58%

cDNA name	Protein name	Legacy name	Number of chromosomes detected	Exon/Intron	Description	Detection method
c.1000C>T	p.Arg334Trp	R334W	1 (0.89^* ^- 4.16 )	Exon 8	C to T at 1132	PCR-RFLP
c.1397C>G	p.Ser466X	S466X	4 (3.57 - 16.66)	Exon 11	C to G at 1529	Sequencing
c.1399C>T	p.Leu467Phe	1531C/T (L467F)	1 (0.89 - 4.16)	Exon 11	C or T at 1531	Sequencing
c.1521-1523delCTT	p.Phe508del	ΔF508	12 (10/71 - 50)	Exon 11	deletion of 3 bp between 1652 and 1655	ARMS and Sequencing
c.1545-1546delTA	p.Tyr515X	1677delTA	4 (3.57 - 16.66)	Exon 11	deletion of TA from 1677	Sequencing
c.1624G>T	p.Gly542X	G542X	1 (0.89 - 4.16)	Exon 12	G to T at 1756	ARMS
c.3909C>G	p.Asn1303Lys	N1303K	1 (0.89 - 4.16)	Exon 24	C to G at 4041	ARMS

^*^ % of all analyzed chromosomes

% of all mutated chromosomes

Alibakhshi *et al* (2008) (13) explored 69 Iranian CF patients sampled from different geographic areas and ethnic groups around Iran. They pointed to a decreasing frequency of p.F508del mutation from North-West to South-East of Europe, considering a gradient which is directed toward Iran. The present study indicated a frequency of approximately 11% (less than 50%) for p.Phe508del emphasizing the previous reports in Iran, (13-15) while the frequency of p.Phe508del is more than 50% in European countries. (18-20) Although most common CFTR mutations in neighboring countries are totally different from what have been recognized in Iran. p.Phe508del still has the highest prevalence in Asian CF patients (21-24).

The p.Tyr515X and p.Ser466X mutations, are both the second most frequent detected mutations in Khorasanian CF patients according to current findings, each found in 3.5% of all examined alleles. The latter mutation indicated to be the second frequent mutation among Iranian CF patients based on Alibakhshi *et al* findings. (13) p.Ser466X is a rare mutation worldwide and was first detected in Southern German patients (25) and has a frequency of approximately 1% in Serbia, Montenegro, and Croatia. (26, 27) 

The p.Asn1303Lys, p.Gly542X, p.Arg334Trp, p.Leu467Phe (L467F) mutations, each had a frequency of approximately 4.1% of all detected mutations and 0.9% of all analyzed chromosomes. p.Asn1303Lys has a high frequency among Mediterranean countries and was reported as the second frequent mutation in Iran, (28) Lebanon (29) and Algeria (30) and third common mutation in Libya (31) and Tunisia. (32) Current findings introduced p.Asn1303Lys as the fourth common detected mutation in North Eastern Iran. The p.Gly542X mutation is distinguished as the second common mutation in CF patients of Spain, (33) Poland (34), Romania (35), and some Spanish origin countries of South America like Costa Roca (36) and Cuba. (37) p.Arg334Trp is more frequent in South American countries and is considered to be associated with greater risk for pancreatitis and renal proteinuria, (38, 39) while the patient carrying this mutation in our study suffered from pancreatic insufficiency and mild proteinuria. p.Leu467Phe is a rare mutation and was first identified in France. 

The mutation that seems to have a different frequency in Khorasan compared to other provinces in Iran is p.Tyr515X /1677delTA. This mutation was the second most frequent mutation among CF patients of Khorasan, while it was 10^th ^common mutation (frequency of about 1%) in Iran according to previous studies. (13) This may refer to different ethnic origin of Khorasanian population. p.Tyr515X is suggested to be considered in the panel of mutations for analysis of CFTR mutations in Khorasan. 

## Conclusion

Current study resulted in identification of seven types of CFTR mutations in 21.42% of CF patients of Khorasan. Regarding the low mutation sensitivity of current study and the great percent of unidentified CFTR mutations (78.58%), it seems to be an unavoidable task to characterize all CFTR mutations by designing more efficient detection methods and whole gene sequencing in future. 

## References

[1] Knowles MR, Durie PR (2002). What is cystic fibrosis?. N Engl J Med.

[2] Riordan JR, Rommens JM, Kerem B, Alon N, Rozmahel R, Grzelczak Z (245). Identification of the cystic fibrosis gene: cloning and characterization of complementary DNA. Science.

[3] Rommens JM, Iannuzzi MC, Kerem B, Drumm ML, Melmer G, Dean M (245). Identification of the cystic fibrosis gene: chromosome walking and jumping. Science.

[4] McCarthy VA, Harris A (2005). The CFTR gene and regulation of its expression. Pediatr Pulmonol.

[5] Zielenski J, Rozmahel R, Bozon D (1991). Genomic DNA sequence of the cystic fibrosis transmembrane conductance regulator (CFTR) gene. Genomics.

[6] Bear CE, Li CH, Kartner N, Bridges RJ, Jensen TJ, Ramjeesingh M (1992). Purification and functional reconstitution of the cystic fibrosis transmembrane conductance regulator (CFTR). Cell.

[7] Zielenski J, Tsui L (1995). Cystic fibrosis: Genotypic and phenotypic variations. Ann Rev Genet.

[8] Cystic Fibrosis Mutation Database.

[9] Estivill X, Bancells C, Ramos C (1997). Geographic distribution and regional origin of 272 cystic fibrosis mutations in European populations. Hum Mutat.

[10] Dawson KP, Frossard PM (2000). The geographic distribution of cystic fibrosis mutations gives clues about population origins. Eur J Pediatr.

[11] Vankeerberghen A, Cuppens H, Cassiman JJ (2002). The cystic fibrosis transmembrane conductance regulator: an intriguing protein with pleiotropic functions. J Cyst Fibros.

[12] Slieker MG, Sanders EAM, Rijkers GT, Ruven HJT, van der Ent CK (2005). Disease modifying genes in cystic fibrosis. J Cyst Fibros.

[13] Alibakhshi R, Kianashirazi R, Jean-Jasques C, Zamani M, Cuppens H (2008). Analysis of the CFTR gene in Iranian cystic fibrosis patients:Identification of eight novel mutations. J Cystic Fibrosis.

[14] Elahi E, Khodadad A, Kupershmidt I, Ghasemi F, Alinasab B, Naghizadeh R (2006). A haplotype framework for cystic fibrosis mutations in Iran. J Mol Diagn.

[15] Jalalirad M, Houshmand M, Mirfakhraie R, Goharbari MH, Mirzajani F (2004). First study of CF mutations in the CFTR gene of Iranian patients: detection of DF508,G542X, W1282X,A120T, R117H, and R347H mutations. J Trop Pediatr.

[16] Esmaeili MR, Akhavan H, Ghabeli A (2011). Detecting common CFTR mutations by reverse dot blot hybridization method in cystic fibrosis first report from Northern Iran. Iran J Pediatr.

[17] Ferrie RM, Schwartz MJ, Robertson NH, Vaudin S, Super M, Malone G (1992). Development, multiplexing, and application of ARMS tests for common mutations in the CFTR gene. Am J Hum Genet.

[18] Guilloud-Bataille M, Crozes DD, Rault G, Degioanni A, Feingold J (2000). Cystic fibrosis mutations: reports from the French registry. Hum Hered.

[19] Onay T, Topaloglu O, Zielenski J, Gokgoz N, Kayserili H, Camcioglu Y (1998). Analysis of the CFTR gene in Turkish cystic fibrosis patients: identification of three novel mutations (3172delAC, P1013L and M1028I). Hum Genet Feb.

[20] Scotet V, Gillet D, Dugue´pe´roux I, Audrezet MP, Bellis G, Garnier B (2002). Spatial and temporal distribution of cystic fibrosis and its mutations in Brittany, France: a retrospective study from 1960. Hum Genet.

[21] Banjar H, Kambouris M, Meyer BF, al-Mehaidib A, Mogarri I (1999). Geographic distribution of cystic fibrosis transmembrane regulator gene mutations in Saudi Arabia. Ann Trop Paediatr.

[22] Eskandarani HA (2002). Cystic fibrosis transmembrane regulator gene mutations in Bahrain. J Trop Pediatr.

[23] Shah U, Moatter T, Bhutta ZA (2006). Profile and factors determining outcome in a cohort of cystic fibrosis patients seen at the Aga khan university hospital, Karachi, Pakistan. J Trop Pediatr.

[24] Rawashdeh M, Manal H (2000). Cystic fibrosis in Arabs: a prototype from Jordan. Ann Trop Paediatr.

[25] Deufel A, Deufel T, Golla A, Achatz H, Bertele-Harms R, Roscher AA (1994). Three novel mutations (I506S, S466X, 1651A→T) in exon 10 of the cystic fibrosis transmembrane conductance regulator (CFTR) detected in patients of southern German descent. Hum Mutat.

[26] Radivojevic D, Djurisic M, Lalic T, Guc-Scekic M, Savic J, Minic P (2004). Spectrum of cystic fibrosis mutations in Serbia and Montenegro and strategy for prenatal diagnosis. Genet Test.

[27] Knezević J, Tanacković G, Matijević T, Barisić I, Pavelić J (2007). Analysis of cystic fibrosis gene mutations and associated haplotypes in the Croatian population. Genet Test.

[28] Alibakhshi R, Zamani M (2006). Mutation analysis of CFTR gene in 70 Iranian cystic fibrosis patients. Iran J Allergy Asthma Immunol.

[29] Farra C, Menassa R, Awwad J, Morel Y, Salameh P, Yazbeck N (2010). Mutational spectrum of cystic fibrosis in the Lebanese population. J Cyst Fibros.

[30] Loumi O, Ferec C, Mercier B, Creff J, Fercot B, Denine R (2008). CFTR mutations in the Algerian population. J Cyst Fibros.

[31] Hadj Fredj S, Fattoum S, Chabchoub A, Messaoud T (2011). First report of cystic fibrosis mutations in Libyan cystic fibrosis patients. Ann Hum Biol.

[32] Fredj SH, Messaoud T, Templin C, des Georges M, Fattoum S, Claustres M (2009). Cystic fibrosis transmembrane conductance regulator mutation spectrum in patients with cystic fibrosis in Tunisia. Genet Test Mol Biomarkers.

[33] Alonso MJ, Heine-Suñer D, Calvo M, Rosell J, Giménez J, Ramos MD (2007). Spectrum of mutations in the CFTR gene in cystic fibrosis patients of Spanish ancestry. Ann Hum Genet.

[34] Kostuch M, Klatka J, Semczuk A, Wojcierowski J, Kulczycki L, Oleszczuk J (2005). Analysis of most common CFTR mutations in patients affected by nasal polyps. Eur Arch Otorhinolaryngol.

[35] Frenţescu L, Brownsell E, Hinks J, Malone G, Shaw H, Budişan L (2008). Study of cystic fibrosis transmembrane conductance regulator gene mutations in a group of patients from Romania. J Cyst Fibros.

[36] Venegas PB, Novak JM, Oscar CA, Sánchez FL, Gutiérrez IG, Rivera JM (2003). Cystic fibrosis mutations in Costa Rica. Hum Biol.

[37] Collazo T, Bofill AM, Clark Y, Hernández Y, Gómez M, Rodríguez F (2009). Common mutations in Cuban cystic fibrosis patients. J Cyst Fibros.

[38] Maisonneuve P, Campbell P, Durie P, Lowenfels AB (2004). Pancreatitis in hispanic patients with cystic fibrosis carrying the R334W mutation. Clin Gastroenterol Hepatol.

[39] Cemlyn-Jones J, Gamboa F (2009). Proteinuria in cystic fibrosis: a possible correlation between genotype and renal phenotype. J Bras Pneumol.

